# Factors Affecting the Use of Social Networks and Its Effect on Anxiety and Depression among Parents and Their Children: Predictors Using ML, SEM and Extended TAM

**DOI:** 10.3390/ijerph192113764

**Published:** 2022-10-22

**Authors:** Evon M. Abu-Taieh, Issam AlHadid, Ra’ed Masa’deh, Rami S. Alkhawaldeh, Sufian Khwaldeh, Ala’aldin Alrowwad

**Affiliations:** 1 Department of Computer Information Systems, Faculty of Information Technology and Systems, The University of Jordan, Aqaba 77110, Jordan; 2 Department Information Technology, Faculty of Information Technology and Systems, The University of Jordan, Aqaba 77110, Jordan; 3 Department of Management Information Systems, School of Business, The University of Jordan, Amman 77110, Jordan; 4 Department Information Technology, Faculty of Information Technology and Systems, University of Fujairah, Fujairah P.O. Box 2202, United Arab Emirates; 5 Department of Business Management, School of Business, The University of Jordan, Aqaba 77110, Jordan

**Keywords:** social networks, TAM, UTAUT, anxiety, depression

## Abstract

Previous research has found support for depression and anxiety associated with social networks. However, little research has explored parents’ depression and anxiety constructs as mediators that may account for children’s depression and anxiety. The purpose of this paper is to test the influence of different factors on children’s depression and anxiety, extending from parents’ anxiety and depression in Jordan. The authors recruited 857 parents to complete relevant web survey measures with constructs and items and a model based on different research models TAM and extended with trust, analyzed using SEM, CFA with SPSS and AMOS, and ML methods, using the triangulation method to validate the results and help predict future applications. The authors found support for the structural model whereby behavioral intention to use social media influences the parent’s anxiety and depression which correlate to their offspring’s anxiety and depression. Behavioral intention to use social media can be enticed by enjoyment, trust, ease of use, usefulness, and social influences. This study is unique in exploring rumination in the context of the relationship between parent–child anxiety and depression due to the use of social networks.

## 1. Introduction

Social networks (SNs) are websites that allow people to interact with each other through their profiles. The participants share comments, publish stories, and interact within the SN by liking /disliking them and commenting. Many types of SN were created for different purposes Facebook, Twitter, LinkedIn, Instagram, Tik-Tok, Sina Weibo, and WeChat, to name a few.

From the user’s perspective, SNs provide numerous benefits, including an e-business forum, creative expression, advertising, a means of communication, keeping up with the news, staying connected with friends, enjoyment, entertainment, amusement, and pastime. Others use SNs for professional networking, such as LinkedIn, as well as for scientific research, such as ResearchGate. Still, negative consequences related to SNs are presented in many studies that relate the use of social networks to depression [1,2,3,4,5,6,7], anxiety [8,9,10], addiction [11,12], loneliness, stress [13,14], mental illness [15], social isolation [16] and many negative connotations as shall be seen in the coming sections.

The goal of this study is to investigate the effect of SN on anxiety and depression in parents and their children within the context of an extended Technology Acceptance Model (TAM) with influencing factors such as perceived usefulness (PU), perceived ease of use (PEoU), perceived enjoyment (EN), social influence (SI), and trust (TR) on behavioral intention (BI). Furthermore, the impact of BI on parents’ anxiety and depression, as well as how this impact relates to children’s anxiety and depression. The research is being carried out in Jordan, an Arabic-speaking country where depression and anxiety are taboo and unspoken.

The motivation of this study is as follows. Many studies were conducted to investigate the negative effects of SN in many cultures, including India [17], USA [1], Spain [6], Scandinavia [2], China [18], and Latinos [14], but only one study was conducted in KSA which studied only depression [19] and in Lebanon which studied addiction [10]. Hence, there is a lack of studies on depression and anxiety in the SN-use arena in the Arabic-speaking world. Further to our knowledge, no study was conducted to show the influence of parents’ depression and anxiety and its influence on children’s anxiety and depression.

The importance of this study tackles a taboo subject in Arabic culture: depression and anxiety among SN users. Such a subject is hardly discussed; one study approached this subject in KSA [19], only tackling depression among adults., Another study was conducted in Lebanon [10] that studied smartphone addiction. Further, the study sheds light on the negative side of SN concerning children. In addition, the study discovers the influence of parents’ depression and anxiety on children’s depression and anxiety.

As for the significance of this contribution, depression and anxiety in SN use are particularly important topics that are not discussed in the Arab-speaking world. We found only two studies in this realm. One pertains to depression and the other to smartphone addiction. No study, as far as we can find, did tackle both topics of depression and anxiety. Hence, there is a big gap to fill. Further, this study extended to the influence of parents’ depression and anxiety on children’s depression and anxiety. As this research will show, there is a high correlation between SN use and parents’ depression and anxiety. Further, there is evidently a high correlation between parents’ depression and anxiety and children’s depression and anxiety. Furthermore, there is a high correlation between enjoyment, usefulness, ease of use, and SN use, despite low trust in SN.

## 2. Literature Review: Anxiety and Depression in SN

Negative effects of SN, such as depression, anxiety, addiction, and social isolation, have been researched in many studies: in [17] the research studied smartphone addiction in India, ref. [20] studied internet addiction, ref. [21] studied TikTok addiction, ref. [22] studied addiction on short films, ref. [11] studied mobile addiction, ref. [12] studied mobile games addiction, and ref. [23] studied Instagram addiction. In addition, ref. [24] studied depression and anxiety severity. While ref. [1,2,18,19] studied depression in Saudi Arabia, China, the USA, and Scandinavia, respectively. While ref. [6] studied depression in tweets in Spain. In fact, studies [3,4,5,15] studied depression. Furthermore, ref. [25] studied the detection of depression using AI. In addition, studies [8,9,26,27] studied depression and anxiety. Additionally, ref. [16] studied social isolation and ref. [10] studied anxiety and smartphone addiction in Lebanon. In [19], the researchers found that 95.4% of the study sample suffered from mild depression to severe depression due to the use of SN on smartphones and found that females are more affected than males. In [18], the research studied depression symptoms among users of WeChat in China. The study concluded “that using social media WeChat showed an association with lower depressive symptoms among people aged ≥45 and older in the study sample.” In [27], the research investigated the role of psychological factors on smartphone addiction proneness (SAP), namely anxiety and low self-control in Korea. In [24], the research studied depression and anxiety symptoms in users of smartphone SN. While ref. [1] studied depressive symptoms among U.S. young adults. In addition, ref. [16] studied perceived social isolation among young adults in the U.S., while ref. [2] reported that 22% of adults suffer from depressive symptoms. In [3], the research proposed a framework for diagnosing user-level depression in SN. While ref. [3] used Facebook ref. [4] used Twitter. In [9], the researchers conducted a systematic review of 70 published studies that discussed social networks (SN) with depression and anxiety. In addition, ref. [5] conducted the same systematic review of research on depression signs on social media. In [15], the study tried to detect depression on SNs by reviewing posts using natural language processing. While ref. [6] detected signs of depression in tweets in Spanish. In [25], the study investigated “Wearable Internet of Medical Things (IoMT) devices with sensors that collect motion data and provide objective measures of physical activity can help to better monitor and detect potential episodes related to the mental health conditions at earlier, more treatable stages”, while ref. [28] did the same using pictures from Instagram. For the purpose of brevity, the following Table 1 shows more than 40 studies that discussed the different negative aspects of SN.

As can be seen in Table 1, none discussed the depression and anxiety of parents and their influence on children, and none discussed the social network behavioral intention in general. None of the studies extended TAM and used the moderators that were suggested by this research. Arabic-speaking cultures were never discussed within the scope suggested by this research. None used SEM, CFA, and ML to validate the results and to build a predictor model.

## 3. Theoretical Framework and Hypotheses Development

**Figure 1:**
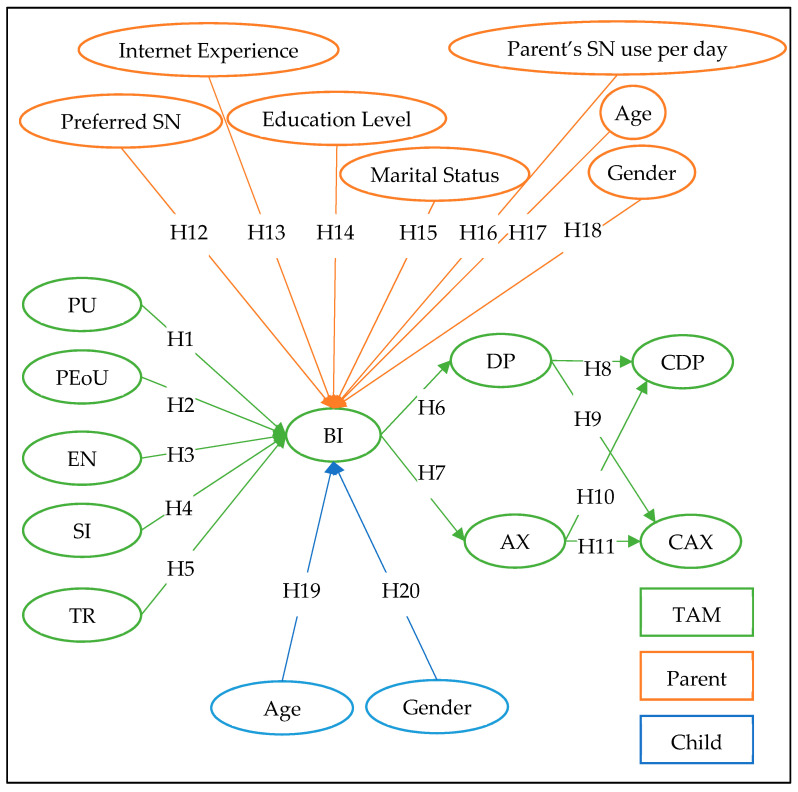
Proposed Model of the Research.

The proposed model is shown in Figure 1, inspired by the original TAM model proposed by Davis et al. [40], in addition to social influence constructs adopted from the TAM extended model [41,42], along with ten moderating factors. The technology acceptance model (TAM) is an information systems theory that models how users come to accept and use a technology. The model’s constructs are adopted from [43,44,45], including perceived usefulness (PU), perceived ease of use (PEoU), perceive enjoyment (EN) as well as social influence (SI), and trust (TR). As the model suggests, the five independent factors influence the parents’ behavioral intention (BI) to use the SN factor [44], which influences the parents’ depression and anxiety factors [46,47], and the effect of parent’s depression and anxiety factors on their children’s depression and anxiety [10,46,47]. The model includes two types of moderators, one pertaining to parents and the second pertaining to children. The parent moderators are age, gender, marital status, education level, internet experience, preferred SN, and time spent on SN. The child moderators are age and gender. The proposed model and hypotheses will be discussed in detail in the following sections. Depression and anxiety were measured using respective 4-item Patient-Reported Outcome Measurement Information System (PROMIS) scales.

The influence of PU, PEoU, EN, TR, and SI on BI are discussed in many studies in many domains. PU’s influence on BI is evident in studies [32,44,48,49,50,51,52,53]. PEoU influence on BI is shown in studies [44,48,51,52,53]. EN influence on BI is also shown in [12,44]. SI influence on BI is studied in [48,54,55] and was used to expand UTAUT. TR is a factor that was used to expand the original UTAUT in [54,55]. Hence, the following hypotheses were postulated.

**Hypothesis** **1** **(H1).** *Perceived usefulness (PU) has a positive influence on parents’ behavioral intention (BI) of using social networks (SN).*

**Hypothesis** **2** **(H2).** *Perceived ease of use (PEoU) has a positive influence on parents’ behavioral intention (BI) of using social networks (SN).*

**Hypothesis** **3** **(H3).** *Perceived enjoyment (EN) has a positive influence on parents’ behavioral intention (BI) of using social networks (SN).*

**Hypothesis** **4** **(H4).** *Social influence (SI) has a positive influence on parents’ behavioral intention (BI) of using social networks (SN).*

**Hypothesis** **5** **(H5).** *Trust (TR) has a positive influence on parents’ behavioral intention (BI) of using social networks (SN).*

The following section will discuss hypotheses related to depression and anxiety and the interplay between parents’ and children’s anxiety and depression.

### 3.1. Anxiety and Depression

Anxiety is “The degree to which an individual’s exposure to and use of a social communication technology induces feelings of tension, worried thoughts, and physical changes like increased blood pressure” [56].

Depression affects how you feel and act. When a person is depressed, s/he may have symptoms such as sadness, hopelessness, or anxiousness; a loss of interest in things s/he once enjoyed; a lack of energy; eating more or less than used to; sleeping too little or too much; trouble thinking or concentrating. Such symptoms should last more than two weeks, and they should not have a medical cause [57].

Anxiety and depression are types of mood disorders. Among other things, depression causes feelings of sadness, hopelessness, and reduced energy. On the other hand, anxiety creates feelings of nervousness, worry, or dread, according to [57].

According to [8], the relationship between anxiety and depression “Several possible models are explored that provide different conceptions of how this relationship may best be understood: (1) that there is a variety of more or less discrete, but sometimes coexisting, syndromes within the spectrum of anxiety and depression; (2) that symptoms of depression and anxiety represent different external manifestations of a more basic underlying cause; (3) that one condition may predispose to the other; (4) that the association may be due to artifactual definitional overlap, particularly since the instruments used to measure depression and anxiety share so many items”.

Further ref. [58] stated that “Depression is a frequent complication of anxiety disorders, and anxiety symptoms are common in primary depressive illness”. The research [46] studied the associations of social media with depression, anxiety, and social isolation. While ref. [10] studied smartphone addiction’s influence on anxiety and problematic family relationships. On the other hand, ref. [33] studied children’s smartphone addiction. Research [31] studied the relationship between Korean parents’ smartphone addiction and that of their children: the mediating effects of children’s depression and social withdrawal. The paper [10] studied the relationship between smartphone addiction, anxiety, and family relations. Even ref. [37] studied developing an empirically sound measure of Facebook-based social support (Facebook Measure of Social Support [FMSS]). While ref. [38] confirmed that there is “a positive correlation between SN use and depression in psychiatric inpatients”. In study [39], depression symptoms among children and adolescents pertaining to SN were discussed. Based on the previous, the following hypotheses were postulated:

**Hypothesis** **6** **(H6).** *Behavioral intention (BI) of parents using social networks (SN) has a positive influence on parents’ depression (DP).*

**Hypothesis** **7** **(H7).** *Behavioral intention (BI) of parents using social networks (SN) has a positive influence on parents’ anxiety (AX).*

**Hypothesis** **8** **(H8).** *The depression of parents (DP) who use social networks has a positive influence on the depression of children (CDP) who use social networks.*

**Hypothesis** **9** **(H9).** *The depression of parents (DP) who use social networks has a positive influence on the anxiety of children (CAX) who use social networks.*

**Hypothesis** **10** **(H10).** *The anxiety of parents (DP) who use social networks has a positive influence on the depression of children (CDP) who use social networks.*

**Hypothesis** **11** **(H11).** *The anxiety of parents (AX) who use social networks has a positive influence on the anxiety of children (CAX) who use social networks.*

### 3.2. Moderators

In this research, there are two types of moderators pertaining to child and parent. The parent moderators are age, gender, educational level, marital status, time spent on the internet, social media venue, and internet experience. On the other hand, moderators of the child are age and gender.

The type of SN that influences behavioral intention was investigated by many [10,11,12,50]. Yang and Gong [11] found that the high quality of games’ interfaces significantly influences smartphone addiction. Ref. [12] stated that perceived enjoyment of a smartphone game is positively associated with smartphone addiction. Sanz-Blas et al. [23] found that overuse and the lack of control over the time spent on SN apps are the main causes of the SN apps addiction. Accordingly, we suggest the following hypothesis:

**Hypothesis** **12** **(H12).** *Parents’ preferred social media has a positive influence on the behavioral intention (BI) of parents using social networks.*

Internet Experience is another important factor that was investigated in [39,54,59,60,61,62,63]. Hence the following hypothesis was postulated.

**Hypothesis** **13** **(H13).** *Parents’ internet experience has a positive influence on behavioral intention (BI).*

Education level is an important factor that influenced behavioral intention in previous studies [54,59,64,65]. Hence the following hypothesis was postulated.

**Hypothesis** **14** **(H14).** *Parent education level has a positive influence on the behavioral intention (BI) of parents using social networks.*

Marital status is one more important moderating factor that was used in other studies, including [54,66,67]. Hence the following hypothesis was proposed.

**Hypothesis** **15** **(H15).** *Parent marital status has a positive influence on the behavioral intention (BI) of parents using social networks.*

Time spent using social media is a moderator factor used in a number of studies such as [55,68,69,70] found that time spent on SN “correlated with psychological distress and suicidal ideation in youth (grades 7–12).” Hence the following hypothesis was proposed:

**Hypothesis** **16** **(H16).** *Parent’s SN use per day has a positive influence on the behavioral intention (BI) of parents using social networks.*

As for age and gender moderators, a number of studies were conducted to investigate age and gender influence on behavioral intention in the information technology arena [54,55,59,60,61,62,66,71,72]. Hence, both the age and gender of the respondent and the child were considered when conducting this research. Based on the previous the following four hypotheses were postulated:

**Hypothesis** **17** **(H17).** *Parents’ age has a positive influence on the behavioral intention (BI) of parents using social networks.*

**Hypothesis** **18** **(H18).** *Parents’ gender has a positive influence on the behavioral intention (BI) of parents using social networks.*

**Hypothesis** **19** **(H19).** *Child age has a positive influence on parents’ behavioral intention (BI) to use social networks.*

**Hypothesis** **20** **(H20).** *Child gender has a positive influence on parents’ behavioral intention (BI) to use social networks.*

### 3.3. Research Questions

This research tries to answer questions as follows: RQ1: What factors influence the Behavioral intention of the parent? Among them are PU, PEoU, EN, SI, and TR. RQ2: which moderators influence the BI? RQ3: Does BI influence the depression and anxiety of the parent? RQ4: Do the parents’ depression and anxiety influence the child’s depression and anxiety? RQ5: Does the parents’ age, gender, internet experience, education level, marital status, and preferred SN influence the behavioral intention to use SN in addition to the child’s age and gender?

## 4. Survey Design & Methods

In this section the research will present survey design and methods. As can be seen in Figure 1 the model is made of 5 independent constructs, three intermediate constructs, and 2 dependent constructs. The independent constructs are PU, PEoU, EN, TR, and SI. The intermediate constructs are behavioral intention (BI), DP, and AX. The dependent constructs are CAX and CDP. There are two types of moderators one pertaining to the parent and the second pertaining to the child. The parent’s moderators are age, gender, marital status, education level, internet experience, preferred SN, and parent’s time spent on SN. The child’s moderators are age and gender. Since research on this issue was limited, the researchers developed a model shown in Figure 1, and in turn, developed the hypotheses above. A questionnaire was developed and assessed, then from a sample of convenience, the data was collected. The next four sections (research context, measurement items, participants and procedure, and measurement instruments) will explain in detail the survey design and method of this research.

### 4.1. Research Context

Many studies were conducted to investigate the relationship between depression and anxiety and SN as shown in Table 1, where more than 40 research were listed pertaining to such an idea but none in the Arab-speaking world. The question raised does the parent’s depression and anxiety influence the child’s depression and anxiety. Hence, this study was conducted as follows.

### 4.2. Measurement Items

A questionnaire survey was created to test the research model proposed for this study. The survey items were developed based on previous research. There are nineteen variables (independent, mediating, dependent, and moderating) in the model. Each variable was measured as follows:

Parents’ age into five groups, gender into two groups, educational level into five groups, Internet experience into three groups, marital status in two groups, child’s age in six groups, time spent on SN in five groups, preferred SN in seven groups, seen in Table A1. The construct PU was measured by five items, and PEoU was measured by four items. EN was measured by three items. Trust (TR) was measured by five items. SI was measured by four items. BI was measured by four items. DP and CDP were measured with four items adopted from [47], and AX and CAX were measured using five items also adopted from [47]. All measurements are shown in Table A1. Next, the participants and collection procedures are presented.

### 4.3. Participants and Procedure

Using Google docs, a web-based survey questionnaire was prepared in both Arabic and English, using a five-point Likert scale ranging from strongly disagree (1) to strongly agree (5). The survey was reviewed by a panel of 11 academicians. Feedback was collected, and the questionnaire was rectified accordingly. Consequently, the survey was piloted on 25 SN users in Jordan to test the understandability of the questions. Revisions were made to the survey.

The survey link was distributed via parents’ school groups, researchers, and Jordan residents on Facebook, LinkedIn, and through schools’ parent WhatsApp groups in Jordan. The convenience sampling method was applied in this study. Participants were voluntary, and no financial incentive was offered.

According to Morgan table data, 384 respondents should be reached for the optimum size of the statistical sample of this research [73]. The survey was conducted from 15 June 2022 to 10 August 2022, and after removing the deficient surveys, 857 SN application users remained. As shown in Table 2, the demographic profile of the respondents for this study showed that the participants were almost equal regarding parents (i.e., males and females), most of them between 28 to less than 38, divorced, holding diploma and bachelor’s degrees, had good and excellent internet experience, spent five hours and more on social media, specifically, on Snapchat and Instagram. Additionally, the demographic profile regarding the children indicated that typically males, between 6 to less than 9 and 12 to less than 15 years old.

### 4.4. Measurement Instruments

The constructs were measured by adopting items from prior well-established instruments with minor wording modifications. A five-point Likert scale, from “strongly disagree” (tagged with 1) to “strongly agree” (tagged with 5), was employed. Table A1 shows the final measurements of constructs.

## 5. Data Analysis and Results

This section presents the results of this study. First, to show the clustering and dispersion of the sample responses, a descriptive analysis was conducted. Second, to test the research hypotheses structural equation model (SEM) was employed with five machine learning methods to validate the results of SEM. Third, the moderators’ effects are presented in this section.

### 5.1. Descriptive Analysis

To describe the responses and thus the attitude of the respondents toward each question they were asked in the survey, the mean and the standard deviation were estimated. While the mean shows the central tendency of the data, the standard deviation measures the dispersion, which offers an index of the spread or variability in the data [74,75]. In other words, a small standard deviation for a set of values reveals that these values are clustered closely about the mean or located close to it; a large standard deviation indicates the opposite. The level of each item was determined by the following formula: (highest point in Likert scale − lowest point in Likert scale)/the number of the levels used = (5 − 1)/5 = 0.80, where 1–1.80 reflected by “very low”, 1.81–2.60 reflected by “low”, 2.61–3.40 reflected by “moderate”, 3.41–4.20 reflected by “high,” and 4.21–5 reflected by “very high”. Then the items were ordered based on their means. Table 3 and Table 4 show the results.

### 5.2. SEM Analysis

SEM analysis was employed to test the research hypotheses. First, Confirmatory factor analysis (CFA) was conducted to ensure the validity and reliability of the observed variables’ responses by calculating Cronbach alpha, composite reliability (CR), and Average Variance Extracted (AVE) for the constructs, and calculating Factor Loadings, Std. Error, Square Multiple Correlation, and Error Variance for every item to each variable. Second, SEM using Amos 20 was performed to test the study hypotheses.

#### 5.2.1. Measurement Model

CFA was conducted to check the properties of the instrument items. Indeed, the measurement model indicates how latent variables or hypothetical constructs are assessed in terms of observed variables; and embodies the validity and reliability of the observed variables’ responses to the latent variables [77,78,79,80]. Table 3 shows the mean, standard deviation, level, order, Cronbach alpha, CR, and AVE for the constructs. Further, Table 4 demonstrates the mean, standard deviation, level, and order scores, Factor Loadings, Std. Error, Square Multiple Correlation, and Error Variance for every item to each variable.

In Table 4, all of the indicators of the factor loadings exceeded the value of (0.50), except one item (PU4 = 0.253) was eliminated to obtain a better fitting measurement model, thus constituting evidence of convergent validity [77,81], Indeed, the measurement reached convergent validity at the item level because all the factor loadings went above 0.50.

In Table 3, all the composite reliability (CR) values exceeded the value of (0.60), demonstrating a high level of internal consistency for the latent variables. In addition, since each value of AVE exceeded the value of (0.50) [77,78], the convergent validity was proved. Further, as presented in Table 3, data analysis results have shown that all research variables are applied to very high levels, whereas the respondent’s attribute of Trust does exist moderately, with a mean of (2.6397).

In addition, as noticed in Table 5, all the intercorrelations between pairs of constructs were less than the square root of the AVE estimates of the two constructs, providing discriminant validity [78]. Consequently, the measurement results indicated that this study had adequate levels of convergent and discriminant validity. Further, the correlation between constructs depending on the absolute value of R can be classified as no relation, weak relation, moderate and strong relation in quarters. Hence, values of less than (0.25) are considered no relation, and values above (0.75) are considered strong relations, while values between [0.25–0.50] are considered weak relations, and values between [0.50–0.75] are considered moderate relations. In the light of the previous one, may read the following:

First, there are 13 strong relationships between the pairs (PU, PEoU), (PEoU, TR), (EN, SI), (SI, AX), (SI, CAX), (TR, BI), (TR, DP), (TR, AX), (TR, CDP), (BI, DP), (BI, AX), (BI, CAX), and (DP, CDP).

Second, there are 13 moderate relationships between the pairs (PU, EN), (PU, BI), (PU, CAX), (PEoU, EN), (EN, DP), (EN, AX), (EN, CDP), (EN, CAX), (SI, CDP), (TR, CAX), (BI, CDP), (DP, AX), and (DP, CAX).

#### 5.2.2. Structural Model

Structural equation modeling using Amos 20 was performed to test the study hypotheses. The results showed that PU, PEoU, EN, SI, and TR positively and significantly impacted BI; thus, H1, H2, H3, H4, and H5 were supported. In addition, BI positively and significantly affected both DP and AX; therefore, H6 and H7 were accepted. DP positively and significantly impacted both CDP and CAX; besides, AX affected both CDP and CAX. Thus, H8-H11 were accepted. Moreover, the coefficient of determination (R²) for the endogenous research variables for BI, DP, AX, CDP, and CAX were (0.752), (0.717), (0.640), (0.698), and (0.488), respectively, which indicates that the model does account for the variation of the proposed model. One can interpret the R² as the proportion of variation in the dependent variable that is predicted by the statistical model. Table 6 provides a summary of the tested hypotheses.

### 5.3. Moderation Effects

As reflected in the model, there are two types of moderators one pertaining to the parent and the second pertaining to the child. The parent moderators are age, gender, marital status, education level, internet experience, preferred SN, and parent’s time spent on SN. The child’s moderators are age and gender. Hypotheses H12–H20 argued that there is a significant difference in the respondents’ BI due to the parent’s age, gender, educational level, marital status, time spent on the internet, preferred SN, internet experience, and child’s age, and gender. ANOVA test was employed to examine if there were any significant differences in the respondent BI that can be attributed to the parent’s age, educational level, time spent on the internet, preferred SN, internet experience, and child’s age. In addition, the Independent Samples *t*-test was employed to investigate if there were any significant differences in the respondent’s BI that can be attributed to the parent’s gender, marital status, and child’s gender.

Results of the ANOVA test, shown in Table 7, indicated that there is a significant difference in the respondents’ BI in favor of parent’s age, educational level, time spent on the internet, preferred SN, internet experience, and child’s age.

Results of the *t*-test, shown in Table 8, indicated that there is a significant difference in the behavioral intention (BI) that can be attributed to parent’s gender, that goes for females more than males. In addition, there is a significant difference in the BI due to the parent’s marital status, which goes for divorced more than married. Further, results indicated that there is a significant difference in the BI that can be attributed to the child’s gender, which goes for females more than males.

### 5.4. ML Validation and Verification of SEM Results

In this section, the research shows that results from SEM can be validated and verified using five ML classification techniques. They are further predicting the influence of DP and AX on CPD and CAX. Modern systems have deployed machine learning techniques as an intelligent technology for decision-making. The techniques have proved their effectiveness in assisting decision-makers in various environments. Therefore, we evaluate five Machine Learning (ML) classification techniques that debrief information from a dataset’s input into a beneficial pattern [82]. Experimentally different ML techniques were selected from various families such as functions, Meta, tree, lazy, and Bayesian techniques. The most effective models that evaluated the depression datasets are Artificial Neural Network (ANN) [83], Linear Regression [84], Sequential Minimal Optimization algorithm for Support Vector Machine (SMO) [85], Bagging using REPTree model [86], and Random Forest [87]. The ANN technique constructs a graph of weights and attached biases to connect the inputs and the target outputs. The training phase uses the back-propagation method to update the weights and bias parameters based on the errors estimated between the predicted and actual output values. The linear regression techniques evaluate the coefficient weights of a linear polynomial function, which are the independent variables (inputs), to reduce the errors of the actual and estimated targets. The SVM techniques depend on vector space to maximize the margin between the targets, whereas evolving updates the values of the weight related to vectors in sequential iterations using the Sequential Minimal Optimization algorithm (SMO). The bagging approach is a member of the Meta family that theorizes that weak learners build strong models (or models). Using a random selection of the instances and features from the training set, REPTree was employed as a weak learner to construct a strong model of significant REPTree models, where the average value of the trees predicts the final value. The Random Forest (RF) is one of the Tree-based models that build an ensemble of a set of decision tree (DT) models. The technique builds the models using samples for each sub-tree model and a random sampling of training data instances. The final output of the model is the average value of the DT trees.

Using a 10-fold cross-validation technique, the models were validated before choosing the optimal version. The 10-fold cross-validation approach is utilized in the evaluation step. This approach chooses 10% of the dataset for testing and 90% for training in a sequential manner (the remaining 9 folds). A classifier model was developed and its effectiveness was evaluated at each step. The average performance is then represented. Hence, reducing the likelihood of over-fitting by using such a strategy, which guarantees that the entire dataset is utilized during the training and testing phases. A difficulty occurs when the model successfully classifies all the training data but is unable to fit the test sets.

#### ML Results and Discussion

The excessive use of social networks leads to two problems, which are depression and anxiety. There are a set of factors that have an impact on children’s depression and anxiety, extending from parents’ anxiety and depression. Hence, a group of datasets was created that relate the input factors to the target variable under study. These datasets are derived from the hypotheses of the studying model in Figure 1, which are (1) dataset 1 incorporates PU, PEoU, EN, SI, and TR (H1-H5) as inputs and BI as output, (2) The BI factor is considered as input with DP (H6) factor to form dataset 2 and with AX (H7) to form dataset 3. (3) The DP and AX are input to CDP (H8, H10) output to form dataset 4, and to CAX (H9, H11) output for dataset 5.

**Figure 2:**
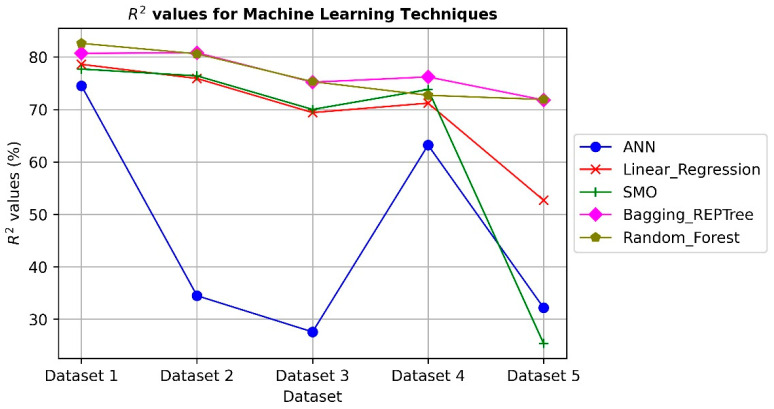
The results of using ML techniques on depression dataset R^2^.

**Figure 3:**
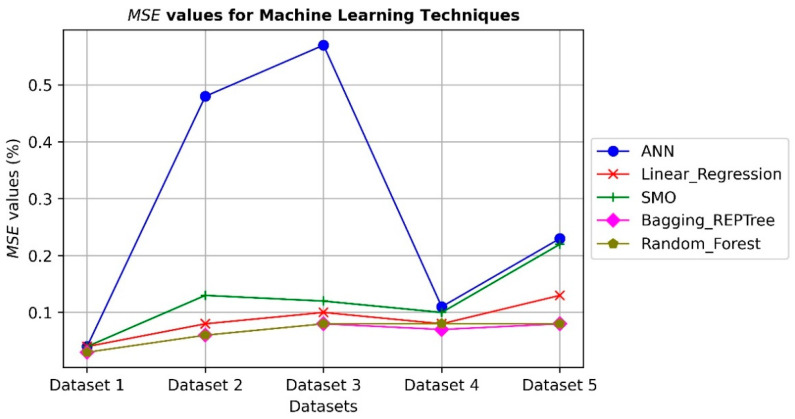
The results of using ML techniques on depression dataset MSE.

ML algorithms, as intelligent procedures, extract inherited important information from datasets to comprehend the relationship between the components (or inputs) and the issues. However, the use of the five datasets to evaluate how well ML models perform. The experimental results are shown in Figure 2 and Figure 3 using R^2^ and Mean Square Error (MSE) as evaluation metrics. The R^2^ and MSE values are on the *y*-axis, and the datasets are on the *x*-axis. The predicted effect of the independent variables on the dependent variable is indicated by the R^2^ statistic (target). The MSE determines the typical divergence between a model’s expected and actual output values.

On five datasets models, the Bagging_REPTree and Random_Forest show reasonable results, with average R^2^ values of 81%. The linear regression and SMO models obtain competitive results reaching up to 75%. The ANN technique provides less performance in comparison with other models due to the divergence of datasets that affects the gradient descent technique during the training phase of the model. The MSE values reflect the error between the actual and predicted values on test sets of the datasets, which have a mirror effect from the R^2^ values. The results indicate the ability of ML techniques, especially the Bagging_REPTree and Random_Forest methods, to predict the children’s depression and anxiety are also extended from the parents’ anxiety and depression when they use social networks.

## 6. Discussion, Implications, and Conclusions

This section will present a discussion of the results, the theoretical and practical implications, and, finally, the conclusion. The section will begin with a discussion of each of the constructs.

The independent construct trust (TR) was of remarkably interesting results. TR was measured with five items: trustworthiness, honesty of SN, Security, protection, and usage trust. In all five items, responders answered very negatively. On the question of trustworthiness, 67% answered disagree, and on the honesty item question 67.1% answered “disagree” to “strongly disagree.” On the security question item, 67.4% disagreed, and 18.2% strongly disagreed. On the fourth item pertaining to information protection of the users, 67% disagreed, and among them 18% strongly disagreed. In the fifth item, which asked, “I trust in social media that I’m using,” we found that 49.2% disagreed while 17.6% strongly disagreed. One may draw a conclusion that although the responders do use SN, yet there is a lack of trust.

**Figure 4:**
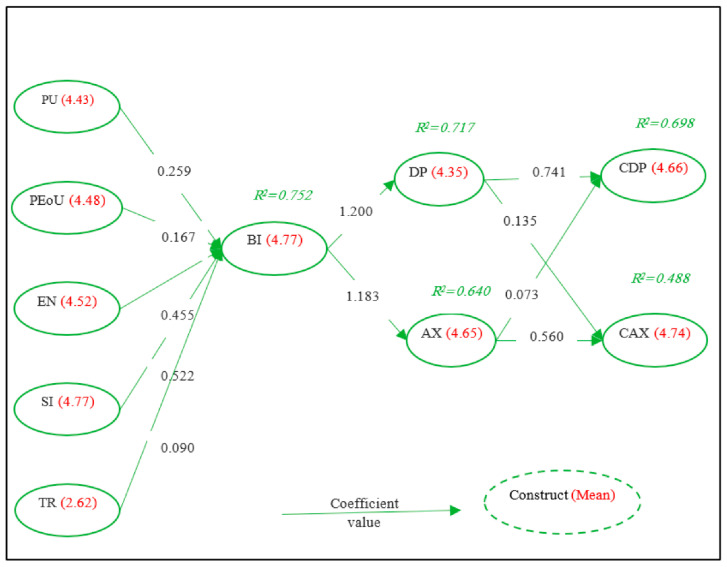
Prosed model of the research with mean, coefficient value, and R-squared.

Figure 4 shows some important results reflected in the proposed model—the mean of each construct, the coefficient value, and the R-squared. The mean gives the central tendency of a set of data; hence the calculated mean of the constructs gives an idea of the tendency of the answers by the respondents. The coefficient value shows how much the dependent variable is expected to increase when that independent variable increases by one. Hence reflecting the strength of the independent variable on the dependent variable. As stated before, one can interpret the R² as the proportion of variation in the dependent variable that is predicted by the statistical model.

Negative side effects, such as depression, anxiety, and addiction, are evident in several studies associated with technology such as smartphones, computer games, and SN, comparable with such studies as [17,20,21,26,29]. Yet people still enjoy and continue to use such technology. In the Enjoyment (EN) constructs, responders answered regarding delightfulness, enjoyability, and entertainment with 96%, 97%, and 97% with “agree” and “strongly agree.” EN construct mean was 4.5, which is an important indicator as the incentive of using SN. As opposed to TR, which is with a mean of (2.6). Responders indicated within their responses that although they may not trust SNs, they enjoy SNs.

Perceived usefulness (PU) was portrayed using five items pertaining to usefulness in daily life, reconnection with people, ease of staying in touch, staying informed, and increased knowledge and education. PU2 and PU4 were the highest in Table 3, with means of 4.77 and 4.94, respectively. The overall mean of the PU construct is 4.4394 and was of order four as shown in Table 2. PU has a strong relationship with PEoU and a moderate relationship with EN, BI, and CAX, as evident in Table 5. PU influence on BI was evident with the support of H1, with a coefficient value of 0.259. Hence, agrees with findings of [43,44,48,49,51,54].

Perceived ease of use (PEoU) was interpreted using four items pertaining to ease of learning, clarity, ease to use, and competency. PU2 and PU3 were the highest in Table 3 with means of 4.76 and 4.77, respectively. The overall mean of the PEoU construct is 4.4837 and was of order three as shown in Table 2. PEoU has a strong relationship with TR and PU, and a moderate relationship with EN shown in Table 5. PEoU influence on BI was evident with the support of H2, with a coefficient value of 0.167. Hence, these results are consistent with findings of [12,48,49,51,54], but not [43,44].

Perceived enjoyment (EN) was interpreted using three items pertaining to delightfulness, enjoyability, and entertainment. EN2 was the highest in Table 3 with a mean of (4.90). The overall mean of the PEoU construct is 4.5235 and was of order two, as shown in Table 2. EN has a strong relationship with SI and a moderate relationship with DP, AX, CDP, CAXPU, and PEoU, as shown in Table 5. EN influence on BI was evident with the support of H3, with a coefficient value of 0.455, which is more than the coefficient values of both PU and PEoU combined. Hence, this is consistent with findings of [12,44,48].

Social Influence (SI) was interpreted using four items pertaining to what people close to the responder think, recommend, and advise, and the fourth is what other people think. S1, S2, and S3 were the highest in Table 3, with means of 4.90, 4.89, and 4.91, respectively. Hence, the closer the people are, the more influence they have. The overall mean of the EN construct is 4.7798 and was of order one, as shown in Table 2. SI has a strong relationship with EN, AX, and CAX, with a moderate relationship with CDP, as seen in Table 5. SI influence on BI was evident with the support of H4, with a coefficient value of (0.522) which is more than the coefficient values of both PU and PEoU combined and EN. Hence, agrees with findings of [48,54,55].

Trust (TR) was investigated using five items pertaining to trustworthiness, honesty, security, protection, and trust. All items were of moderate level. The highest was TR2, and the lowest was TR3. Hence responders doubt the honesty and security of SN. In fact, the security item was the lowest compared with all items in the questionnaire. The whole construct means, as shown in Table 3, had the lowest mean (2.6397). There is a strong relationship between TR and BI, DP, AX, CDP, and PEoU, with a moderate relationship with CAX, as seen in Table 5. BI was evident with the support of H5, with a coefficient value of 0.090. Hence, it agrees with the findings of [44,48].

The behavioral intention (BI) construct was measured by four items pertaining to future use, whenever I can, planning to use in the future, and recommending to others. BI2, BI3, and BI4 were the highest in Table 4, with means of 4.91, 4.91, and 4.87, respectively. The overall mean of the BI construct is 4.7748 and was of order one, as shown in Table 3. BI has a strong relationship, according to Table 5, with TR, DP, AX, CAX, and moderate relation with CDP and PU. Further, according to findings in Table 7 and Table 8 that there is a significant difference in the BI attributed to parent gender (female), parent marital status (divorced), and child gender (female). In addition, according to findings seen in Table 6, BI was positively influenced by PU, PEoU, EN, SI, and TR where H1–H5 were supported. Moreover, BI positively influences DP and AX where H6- and H7 were supported with coefficient values of 1.200 and 1.183, respectively. This agrees with the findings of [10].

The parent’s anxiety (AX) construct was measured by four items pertaining to uneasiness, nervousness, calming down, and anxiousness. The highest were AX2 and AX4 in Table 4, which is an item asking nervousness item with a mean of 4.84 and 4.83, respectively. The overall mean of the AX construct is 4.6511 and was of order two, as shown in Table 3. As seen in Table 5, AX had a strong relationship with SI, TR, and BI while it had a moderate relationship with EN and DP. AX, as a mediator construct in the proposed model, was influenced by BI according to the supported H7. In fact, the coefficient value was 1.183. and influenced CDP and CAX with coefficient values of (0.073) and (0.560), respectively. Which agrees with findings of [10,31,34,57,58].

The parent’s depression (DP) construct was measured by six items pertaining to cheer up, unhappiness, interest, loneliness, upset, and depression. DP3, DP4, and DP5 were the highest in Table 4 with means 4.82, 4.84, and 4.82, respectively. This entails that the responder uses SN to reduce unhappiness, to make life interesting, and so they would not feel lonely. The overall mean of the DP construct is 4.3587 and was of order three, as shown in Table 3. DP had a strong relationship with TR, and BI, as shown in Table 5, while having a moderate relationship with EN and a weak relationship with SI. DP was influenced by BI according to the supported H6 with a coefficient value of 1.200. And DP did influence CDP and CAX with coefficient values of 0.741 and 0.135, respectively. Which agrees with findings of [10,31,57,58]. The study [38] stated that there is “a positive correlation between SN use and depression in psychiatric inpatients,” which agrees with the finding of H6.

The child’s depression (CDP) construct was measured by six items pertaining to cheer up, unhappiness, interest, loneliness, upset, depressed. The highest items were DP1, DP2, and DP6 with means of 4.86, 4.87, and 4.86, respectively. The overall mean of the CDP construct is 4.6622 and was of order two, as shown in Table 3. CDP had a strong relationship with TR and DP, as seen in Table 5, with a moderate relationship with EN, SI, and BI. CDP was positively influenced by DP and AX according to the supported H8, and H10 with coefficient values of 0.741 and 0.073. This agrees with the findings of [10,31,34].

The child’s anxiety (CAX) construct was measured by four items pertaining to uneasiness, nervousness, calming down, and anxiousness. The highest were CAX2 and CAX3 with means (4.87) and (4.88) respectively. The overall mean of the CAX construct is (4.7447) and was of order one as shown in Table 3. CAX had a strong relationship with SI and BI as seen in Table 5, with a moderate relationship with PU, EN, and BI. CAX was positively influenced by DP and AX according to the supported H9, and H11 with coefficient values of 0.135 and 0.560, respectively, which agrees with findings from [10,12,23,31,34].

The moderators that affected BI were parents’ gender (female), parents’ marital status (divorced), and child’s gender (female). Further, there was a significant difference in the respondent BI in favor of parents’ age, parents’ educational level, parents’ time spent on the internet [38], parents’ preferred SN, parents’ internet experience, and child’s age. Age and gender agree with the finding of [54,61], time spent agrees with the findings of [55,70] and disagree with [38].

In conclusion, the research found that BI is influenced by all five independent constructs which are EN, TR, PU, PEoU, and SI. Additionally BI, the intermediate construct, influences parents’ depression and anxiety. Both parents’ depression and anxiety influence both child’s depression and anxiety. Further, BI to use SN is influenced by moderators: parents’ and child’s gender, and parents’ marital status towards female and divorced, respectively. In addition, other moderators did have an influence on BI including parents’ age, educational level, time spent on the internet, preferred SN, internet experience, and child’s age.

### 6.1. Theoretical Implications

In this section, the research presents the theoretical implications. First, social networks (SN) are a new ecosystem with both positive and negative aspects. As a result, this research area is underserved. As a result, this study will serve as a springboard for further investigation by researchers, practitioners, educators, and parents.

Second, the findings are consistent with the arguments of social network analysis and its relation to depression and anxiety of both parents and children, as shown in the previous section.

Third, as shown above, the gender of parent and child, as well as marital status had a significant difference in favor of female and divorced status; such findings must be investigated further.

Fourth, there is an obvious conflict between enjoyment and trust. Hence people have a lack of trust in SNs, yet they enjoy the SNs. Further, social influence (SI) is a major factor that influences user behavior. This is understandable since the word of mouth and the peer pressure to join certain SN.

### 6.2. Practical Implications

As for the practical implications of this research, they are as follows: this study was successfully able to provide further understanding regarding this important problem related to the negative aspects of SN. Knowing that SNs are new technology, hence developers may include warnings for users regarding time spent. Further psychological evaluation tests can be developed to detect depression and anxiety symptoms.

In addition, this study provides empirical evidence about the key dimensions that should be considered by researchers and SN developers and their social and moral responsibilities towards SN users. Further, SN users should be alert to such claims that associate SN use with anxiety and depression.

The high correlation shown in this study between the use of behavioral intention (BI) with parents’ depression and anxiety does not mean causation, but it does require more in-depth research. Further, such a finding must be a wake-up call to SN users and designers and developers. Further, the high correlation between parents’ depression and anxiety with the child’s depression needs to be investigated.

Some suggestions can be made regarding the long hours while using SN is to have an alarm that will alert the SN user. Hence, a framework for depression and anxiety detection software can alert parents and children regarding their depression and anxiety.

Another practical implication is the rating of SN, whether it is fit for kids or not, as with the movies, rather than leaving such matters in the hands of industry. Views and likes motivate the user.

As SN is a new culture, more education must be provided for parents through schools and SN itself. Adults should be aware of the negative effects of SN to protect themselves and their children.

### 6.3. Limitations and Directions for Further Research

Many difficulties were faced in this research. First, Jordanian and most Arab communities are very reserved when talking about topics such as depression and anxiety. In fact, people consider such topics taboo and prefer not to discuss them. Hence, the questionnaire promised anonymity, and the data will be used only as part of scientific research. Therefore, it was hard to collect data about respondents and their children. Further, respondents may try to protect their children from being stigmatized.

Second, there was an underrepresentation of parents over 48, which is understandable since we were targeting parents with children less than 17 years old, and parents’ educational level of Ph.D. and high school or less. In addition, the children’s gender was mainly male 69.4%. Of the marital status of parents, the divorced only constituted 34.4%. Parents’ preferred SN was Snapchat and Instagram, 47.7% and 30%, respectively. Children’s ages included 0–3, 3–6, 9–12, and 15–17, but the majority were 6–9 (65%) and 12–15 (28.4%).

Third, SNs are constantly developing and changing. Hence, to measure one SN and its relation to depression and anxiety is almost impossible. Still, there is an obvious need to develop a constant measure with the help of information technology and psychiatry professionals that help in the diagnosis of the initial stages of depression and anxiety.

Another difficulty faced is that SN does not share data with researchers. Given the publicly available data, it does not provide the researchers with the essential data needed to analyze the parent’s depression and anxiety and its influence on the children’s depression and anxiety.

Still, other areas of investigation, such as word of mouth and peer pressure are other aspects that should be investigated in this arena. Especially from the perspective of the parent. A high correlation between parents’ depression and anxiety with the child’s depression but not with the child’s anxiety was noticed. As such, this relationship should be investigated further.

Additionally, because these findings were correlational rather than predictive, the researchers were only able to demonstrate the existence of a trend between the variables and were unable to create a model that would predict how they would interact.

### 6.4. Conclusions

This study used an extended model TAM to study the influence of parents’ anxiety and depression on children’s anxiety and depression when using SNs. The research used CFA, SEM, and five ML methods to validate and verify the findings as in [88,89,90,91]. The research found that there is a high correlation between parents’ depression and anxiety with children’s depression and a weak correlation with children’s anxiety. In addition, it was found that although parents enjoy the use of SNs, they do not trust SNs. There is a high correlation between ease of use and usefulness with the use of SNs. There is a difference between divorced vs. married in favor of divorce, and gender in favor of females when using SNs.

The study did support conclusions from many studies. PU influence on BI agrees with findings of [43,44,48,49,51,54]. PEoU influence on BI agrees with findings of [12,48,49,51,54], but not [43,44]. EN influence on BI consistent with findings of [12,44,48]. TR’s influence on BI agrees with findings of [44,48]. BI influences on DP and AX agree with the findings of [10]. AX influence on CAX agrees with the findings of [10,31,34,57,58]. DP influence on CAX and CDP agrees with the findings of [10,31,34,57,58]. The moderators’ influence on BI, as explained previously, was supported by many studies, including [54,55,61,70].

## Figures and Tables

**Table 1:** Studies pertain to addiction, depression, and anxiety in using SN.

Source	Topic	Factors (Country)
[17]	Smartphone addiction	Loneliness, stress, depression, ease of use, cost (India)
[29]	Smartphone addiction	Features of smartphone
[20]	Internet addiction	CIUT extend UGT
[26]	Role of rumination	CIUT extend UGT: depression, social anxiety, stress
[21]	Addiction behavior in short films (TIKTOK)	Opponent process theory (OPT)
[22]	Addiction behavior in short films	Socio-technical approach and attachment theory: social interaction anxiety, neuroticism and social isolation
[24]	Depression & anxiety severity	In SN
[19]	Depression	Patient Health Questionnaire 9 (PHQ-9) (Saudi Arabia)
[18]	Depression	WeChat, depression symptoms (China)
[27]	Anxiety	Behavioral Inhibition System (BIS) and Behavioral Activation System (BAS) scales (Korea)
[1]	Depression	Problematic social media use (PSMU) (USA)
[2]	Digital depression	New disease (Scandinavia)
[3]	Depression	Facebook data mining, AI
[4]	Depression	Machine learning, AI
[5]	Depression	Detecting depression signs in social media
[15]	Depression and mental illness	Natural language processing methods
[6]	Depression in tweets in Spanish	Linguistics analysis (Spanish)
[25]	Detection of depression	AI
[16]	Social isolation	Statistical analysis of using both time and frequency (KSA)
[19]	Adoption of internet	Extended TAM (KSA)
[7]	Depression	Russian language mental health app (Russia)
[8]	Depression and anxiety	Relationship between anxiety and depression
[9]	Depression and anxiety	Social networking sites, depression, and anxiety
[11]	Mobile addiction	Addiction, cost and affordance, SOR theory
[12]	Mobile game addiction	Perceived visibility, perceived enjoyment and flow
[23]	Instagram addiction	Overuse and stress
[13]	Depression, anxiety, or stress	Twitter, ML, AI
[14]	SN use and depression, anxiety, and stress	Predictions using regression (Latinos)
[30]	Depression predictor (software)	ML and TAM and facial Features
[31]	Parent’s addiction to smartphone	
[32]	Satisfaction with Web-based information services (WIS)	TAM and uses and gratifications theory (U&G)
[10]	Smartphones	Addiction and Anxiety and problematic family relations
[33]	Smartphone addiction	Studied children smartphone addiction
[31]	Parents’ smartphone addiction	The Mediating Effects of Children’s Depression and Social Withdrawal (Korea)
[10]	Smartphone addiction, anxiety	Family relations (Lebanon)
[34]	Internet addiction	Depression, hostility, and social anxiety
[35]	SN use	Depression
[36]	SN	Depression and anxiety (USA)
[37]	To develop (Facebook Measure of Social Support [FMSS])	Depression—adolescence
[38]	Relationship between social support and depression	The effect of social networking sites on the relationship between perceived social support and depression
[39]	SN	Depression symptoms—child and adolescent

**Table 2:** Description of the respondents’ demographic profiles.

Category	Category	Frequency	Percentage%
Parent’s Gender	Male	435	50.8
	Female	422	49.2
	Total	857	100
Parent’s Age (Year)	18 to less than 28	51	6.0
	28 to less than 38	650	75.8
	38 to less than 48	154	18.0
	48 to less than 58	2	0.2
	58 and over	0	0
	Total	857	100
Parent’s Educational Level	High school or less	0	0
	Diploma	405	47.3
	Bachelor’s	300	35.0
	Master	150	17.5
	Ph.D.	2	0.2
	Total	857	100
Parent’s Marital Status	Married	554	64.6
	Divorced	303	34.4
	Total	857	100
Child’s Gender	Male	595	69.4
	Female	262	30.6
	Total	857	100
Child’s Age (Year)	From less than 1 to less than 3	22	2.6
	3 to less than 6	14	1.6
	6 to less than 9	558	65.1
	9 to less than 12	12	1.4
	12 to less than 15	243	28.4
	15 to less than 17	8	0.9
	Total	857	100
Parent’s Internet Experience	Low	5	0.6
	Good	675	78.8
	Excellent	177	20.7
	Total	857	100
Parent’s SN Use per Day (Hours)	One	5	0.6
	Two	15	1.8
	Three	419	48.9
	Four	12	1.4
	Five and more	406	47.4
	Total	857	100
Parent Preferred SN	Facebook	21	2.5
	Twitter	1	0.1
	TikTok	7	0.8
	Snapchat	409	47.7
	LinkedIn	0	0
	YouTube	9	1.1
	Instagram	257	30.0
	Other	153	17.9
	Total	857	100

**Table 3:** Overall mean and standard deviation of the study’s variables.

Type of Variable	Variables	Mean	Standard Deviation	Level	Order	Cronbach Alpha	CR *	AVE **
**Independent Variables**	Perceived Usefulness (PU)	4.4394	0.19185	Very High	4	0.889	0.9	0.91
	Perceived Ease of Use (PEoU)	4.4837	0.14808	Very High	3	0.854	0.95	0.96
	Perceived Enjoyment (EN)	4.5235	0.44154	Very High	2	0.806	0.9	0.75
	Social Influence (SI)	4.7798	0.39137	Very High	1	0.871	0.93	0.94
	Trust (TR)	2.6397	1.17887	Moderate	5	0.981	0.94	0.95
**Mediating Variable**	Behavioral Intention (BI)	4.7748	0.41038	Very High	1	0.848	0.89	0.91
	Parent Depression (DP)	4.3587	0.56172	Very High	3	0.951	0.95	0.76
	Parent Anxiety (AX)	4.6511	0.58048	Very High	2	0.882	0.93	0.78
**Dependent Variable**	Child Depression (CDP)	4.6622	0.52786	Very High	2	0.917	0.94	0.95
	Child Anxiety (CAX)	4.7447	0.53278	Very High	1	0.927	0.95	0.96

* Employing Fronell and Larcker’s [76] formula. ** The formula for the variance extracted. CR: Composite Reliability.

**Table 4:** Properties of the final measurement model.

Construct and Items	Mean	SD	Level	Order	Factor Loadings	Std. Error	Square Multiple Correlation	Error Variance
**Perceived Usefulness (PU)**								
PU1	4.11	0.693	High	5	0.685	***	0.469	0.255
PU2	4.77	0.443	Very High	2	0.76	0.034	0.577	0.38
PU3	4.19	0.435	High	3	0.926	0.034	0.857	0.27
PU4	4.94	0.263	Very High	1				
PU5	4.18	0.444	High	4	0.918	0.034	0.843	0.31
**Perceived Ease of Use (PEoU)**								
PEoU1	4.2	0.414	High	3	0.951	***	0.904	0.16
PEoU2	4.76	0.445	Very High	2	0.807	0.025	0.651	0.169
PEoU3	4.77	0.435	Very High	1	0.831	0.024	0.691	0.158
PEoU4	4.2	0.42	High	3	0.953	0.016	0.908	0.116
**Perceived Enjoyment (EN)**								
EN1	4.24	0.545	Very High	3	0.94	***	0.81	0.24
EN2	4.9	0.417	Very High	1	0.51	0.021	0.261	0.128
EN3	4.43	0.584	Very High	2	0.701	0.025	0.491	0.174
**Social Influence (SI)**								
SI1	4.9	0.402	Very High	2	0.92	***	0.846	0.25
SI2	4.89	0.448	Very High	3	0.948	0.022	0.898	0.2
SI3	4.42	0.579	Very High	4	0.519	0.048	0.27	0.244
SI4	4.91	0.391	Very High	1	0.94	0.02	0.884	0.18
**Trust (TR)**								
TR1	2.64	0.927	Moderate	3	0.942	***	0.888	0.096
TR2	2.9	1.348	Moderate	1	0.941	0.025	0.886	0.207
TR3	2.45	1.12	Moderate	5	0.986	0.017	0.972	0.36
TR4	2.74	1.513	Moderate	2	0.988	0.023	0.975	0.56
TR5	2.46	1.114	Moderate	4	0.991	0.017	0.981	0.23
**Behavioral Intention (BI)**								
BI1	4.41	0.624	Very High	3	0.517	***	0.267	0.285
BI2	4.91	0.411	Very High	1	0.893	0.069	0.797	0.34
BI3	4.91	0.395	Very High	1	0.867	0.065	0.751	0.39
BI4	4.87	0.517	Very High	2	0.951	0.09	0.905	0.25
**Parent Depression (DP)**								
DP1	4.17	0.7	High	3	0.739	***	0.546	0.222
DP2	3.9	0.482	High	5	0.906	0.03	0.82	0.42
DP3	4.82	0.682	Very High	2	0.977	0.041	0.954	0.21
DP4	4.84	0.654	Very High	1	0.962	0.04	0.926	0.32
DP5	4.82	0.691	Very High	2	0.981	0.042	0.963	0.18
DP6	4.08	0.638	High	4	0.744	0.04	0.553	0.182
**Parent Anxiety (AX)**								
AX1	4.38	0.67	Very High	4	0.632	***	0.399	0.269
AX2	4.84	0.636	Very High	1	0.97	0.063	0.94	0.24
AX3	4.56	0.725	Very High	3	0.774	0.067	0.598	0.211
AX4	4.83	0.668	Very High	2	0.989	0.066	0.978	0.1
**Child Depression (CDP)**								
CDP1	4.86	0.587	Very High	2	0.972	***	0.945	0.19
CDP2	4.87	0.57	Very High	1	0.977	0.011	0.954	0.15
CDP3	4.39	0.679	Very High	5	0.667	0.031	0.445	0.255
CDP4	4.59	0.674	Very High	3	0.721	0.029	0.519	0.218
CDP5	4.4	0.655	Very High	4	0.62	0.032	0.385	0.264
CDP6	4.86	0.592	Very High	2	0.94	0.015	0.883	0.41
**Child Anxiety (CAX)**								
CAX1	4.61	0.626	Very High	3	0.974	***	0.949	0.2
CAX2	4.87	0.549	Very High	2	0.702	0.023	0.493	0.153
CAX3	4.88	0.532	Very High	1	0.699	0.022	0.489	0.145
CAX4	4.61	0.638	Very High	3	0.982	0.011	0.964	0.15

*** Zero Value.

**Table 5:** Correlations of constructs.

Constructs	PU	PEoU	EN	SI	TR	BI	DP	AX	CDP	CAX
**PU**	**0.95**									
**PEoU**	0.915	**0.98**								
**EN**	0.529	0.503	**0.97**							
**SI**	0.317	0.319	0.775	**0.97**						
**TR**	0.310	0.790	0.480	0.383	**0.87**					
**BI**	0.530	0.103	0.310	0.430	0.809	**0.95**				
**DP**	0.160	0.113	0.680	0.335	0.858	0.798	**0.88**			
**AX**	0.270	0.125	0.700	0.892	0.809	0.940	0.614	**0.87**		
**CDP**	0.229	0.115	0.671	0.522	0.810	0.592	0.880	0.372	**0.98**	
**CAX**	0.700	0.460	0.680	0.797	0.552	0.862	0.673	0.330	0.330	**0.97**

Note: Diagonal elements are square roots of the average variance extracted for each of the ten constructs. Off-diagonal elements are the correlations between constructs.

**Table 6:** Summary of proposed results for the theoretical model.

Research Proposed Paths	Coefficient Value	*t*-Value	*p*-Value	Empirical Evidence
H1: PU → BI	0.259	8.493	0.000	Supported
H2: PEoU → BI	0.167	4.053	0.000	Supported
H3: EN → BI	0.455	32.950	0.000	Supported
H4: SI → BI	0.522	33.499	0.000	Supported
H5: TR → BI	0.090	17.440	0.000	Supported
H6: BI → DP	1.200	46.548	0.000	Supported
H7: BI → AX	1.183	38.980	0.000	Supported
H8: DP → CDP	0.741	30.524	0.000	Supported
H9: DP → CAX	0.135	4.141	0.000	Supported
H10: AX → CDP	0.073	3.133	0.000	Supported
H11: AX → CAX	0.560	17.979	0.000	Supported

**Table 7:** ANOVA Analysis of respondent Behavioral Intention (BI) attributed to parent age, parent educational level, parent time spent on the internet, parent’s preferred SN, parent’s internet experience, and children’s age.

Variable		Sum of Squares	Df	Mean Square	F	Sig.
BI attributed to *parent age*	Between Groups	62.831	11	5.712	35.280	0.000
	Within Groups	136.809	845	0.162		
	**Total**	**199.641**	**856**			
BI attributed to *parent educational level*	Between Groups	389.652	11	35.423	299.82	0.000
	Within Groups	99.834	845	0.118		
	**Total**	**489.487**	**856**			
BI attributed to *parent time spent on the internet*	Between Groups	822.713	11	74.792	611.438	0.000
	Within Groups	103.362	845	0.122		
	**Total**	**926.075**	**856**			
BI attributed to *Parent preferred SN*	Between Groups	2451.675	11	222.880	388.823	0.000
	Within Groups	484.367	845	0.573		
	**Total**	**2936.042**	**856**			
BI attributed to *parent internet experience*	Between Groups	33.651	11	3.059	22.710	0.000
	Within Groups	113.828	845	0.135		
	**Total**	**147.480**	**856**			
BI attributed to *child age*	Between Groups	378.299	11	34.391	54.988	0.000
	Within Groups	528.481	845	0.625		
	**Total**	**906.779**	**856**			

**Table 8:** *t*-test of the respondent Behavioral Intention (BI) attributed to parent gender, parent marital status, and child gender.

Variable	Parent Gender: Male			Parent Gender: Female			*t*	df	Sig.
	N	Mean	Std. Dev.	N	Mean	Std. Dev.			
BI	435	4.6799	0.32061	422	4.8726	0.46643	7.029	743.802	0.000
	**Parent Marital Status: Married**			**Parent Marital Status: Divorced**			***t***	**df**	**Sig.**
BI	554	4.6606	0.45269	303	4.9835	0.18573	14.679	805.975	0.000
	**Child Gender: Male**			**Child Gender: Female**			***t***	**df**	**Sig.**
BI	595	4.7290	0.38460	262	4.8788	0.44728	4.709	438.205	0.000

## Data Availability

Collected responses is available upon request.

## Appendix A

**Table A1:** Constructs and items.

Constructs	ID: Items/Measure	Adopted from
Demographic Information	Parent Gender 1: Male. 2: Female.	[55]
	Parent Age (years) 1: 18 to less than 28. 2: 28 to less than 38 years old. 3: 38 to less than 48 years old. 4: 48 to less than 58 years old. 5: Over 58 years old.	[55]
	Parent Educational level 1. High school and less. 2: Diploma. 3: Bachelor. 4: Master. 5: Ph.D.	[92]
	Marital Status 1: Divorced. 2: Married.	[54,66]
	Child Gender 1: Male. 2: Female.	[55]
	Child Age (years) 1: From less than 1 to less than 3. 2: 3 to less than 6 years old. 3: 6 to less than 9 years old. 4: 9 to less than 12 years old. 5: 12 to less than 15 years old. 6: 15 to less than 17 years old.	[55]
	Parent Internet experience 1: Low. 2: Good. 3: Excellent.	[93]
	Time spent on social media (hours) 1: one. 2: two. 3: three. 4: four. 5: Five and more.	[55]
	Which of the following is your favorite social network that you are using most of the time? 1: Facebook. 2: Twitter. 3: TikTok. 4: Snapchat. 5: LinkedIn. 6: YouTube. 7: Instagram. 8: Other.	
Perceived usefulness (PU)	**PU1**: social media is useful in my daily personal life. **PU2**: Using social media enables me to get re-connected with people that matter to me. **PU3**: Using social media makes it easier to stay in touch with others effectively. **PU4**: Using social media makes it easier to stay informed with my friends and family. **PU5**: Using social media increases my educational, knowledge, and cultural levels.	[32,44,48,49,50,51,52,53]
Perceived ease of use (PEoU)	**PEoU1**: Learning how to use social media is easy for me. **PEoU2**: My interaction with social media is clear and understandable. **PEoU3**: social media is easy to use. **PEoU4**: It is easy for me to become skillful at using social media.	[44,48,51,52,53]
Perceived enjoyment (EN)	**EN1**: I feel that using social media is delightful. **EN2**: I feel that using social is enjoyable. **EN3**: I feel that using social is very entertaining.	[48,54,55]
Trust (TR)	**TR1**: I believe that social media is trustworthy, so I use them. **TR2**: I do not doubt the honesty of social media. **TR3**: social media provides security for my information. **TR4**: I feel assured that legal and technological structures adequately protect me from problems of using social media. **TR5**: I trust in social media that I’m using.	[94]
Social Influence (SI)	**SI1**: People I know, think that I should use social media. **SI2**: People who are important to me would recommend that I use social media. **SI3**: People, who are important, think that I should use social media. **SI4**: Everyone around me is thinking that I should use social media because they are using it.	[55,95]
Behavioral Intention (BI)	**BI1**: I intend to use social media in the future. **BI2**: I am using social media and I always try to use it whenever I can at any time. **BI3**: I plan to keep using social media in the future. **BI4**: I will recommend others to use social media.	[55,94]
Parent—Anxiety (AX)	**AX1**: I feel uneasy when I do not use social media. **AX2**: I feel nervous when I do not use social media. **AX3**: I have difficulty calming down when I do not use social media. **AX4**: I felt anxious when I do not use social media.	[47]
Parent—Depression (DP)	**DP1**: I feel that nothing could cheer me up except when I’m using social media. **DP2**: I feel unhappy when I’m not using social media. **DP3**: I feel that nothing was interesting except using social media. **DP4**: I feel lonely when I’m not using social media. **DP5**: I feel upset for no reason when I’m not using social media. **DP6**: I feel depressed when I’m not using social media.	[47]
Child—Anxiety (CAX)	**CAX1**: my child feels uneasy when he/she does not use social media. **CAX2**: my child feels nervous when he/she does not use social media. **CAX3**: my child has difficulty calming down when he/she does not use social media. **CAX4**: my child feels anxious when he/she does not use social media.	[47]
Childe Depression (CDP)	**CDP1**: my child feels that nothing could cheer him/her up except when he/she is using social media. **CDP2**: my child feels unhappy when he/she is not using social media. **CDP3**: my child feels that nothing was interesting except using social media. **CDP4**: my child feels lonely when he/she is not using social media. **CDP5**: my child feels upset for no reason when he/she is not using social media. **CDP6**: my child feels depressed when he/she is not using social media.	[47]
